# Effects of ascorbic acid and cysteine supplementation on preimplantation embryo development and oxidative stress-related gene expression in dromedary camels

**DOI:** 10.1186/s12917-025-05181-1

**Published:** 2025-12-18

**Authors:** Ahmed Mohamed Kamel, Nasser Ghanem, Gamal Ashour, Khalid Ahmed El-Bahrawy

**Affiliations:** 1 Animal and Poultry Production Division, Desert Research Center, Cairo, 11753 Egypt; 2 Department of Animal Production, Faculty of Agriculture, Cairo University, Giza, Egypt

**Keywords:** In vitro, Embryos, Dromedary, Culture medium, Antioxidants

## Abstract

**Background:**

Oxidative stress (OS) is a harmful threat during early preimplantation that compromises embryonic development. Ascorbic acid and cysteine were found to have significant contributions in various physiological processes, including embryonic development and mitigating various stressors, by acting as antioxidants and regulating gene expression. This study evaluated the mitigating impact of ascorbic acid and cysteine addition on OS during in vitro culture (IVC) and subsequent early embryonic developmental stages of the dromedary camel. The ovaries were sourced from a nearby slaughterhouse; only high-quality oocytes were used for in vitro embryo production (IVP). Produced zygotes were in vitro cultured with ascorbic acid, cysteine, or both under a high oxygen level (20%). This study included four experimental groups: an untreated group without antioxidant i.e., control (T1), the 2nd group supplemented with 150 µg/mL ascorbic acid (T2), the 3rd group supplemented with 100 µM cysteine (T3), and the 4th group (T4) enriched with a combination of both antioxidants (150 µg/mL ascorbic acid and 100 µM cysteine). Embryo development was monitored throughout different preimplantation stages. Real-time PCR was used to assess the relative abundance of various genes, including genes that are related to oxidative stress (catalase (CAT), superoxide dismutase (SOD), and thioredoxin (TXN)), apoptosis related genes (B-cell lymphoma 2 (BCL2), and tumor suppressor protein (p53)), and metabolic related gene (glucose transporter 1 (GLUT-1)).

**Results:**

The results indicated an increased cleavage rate (*P* < 0.05) in T2 (29.41%), T3 (32.77%), and T4 (27.16%) compared to T1 (14.05%). Moreover, the rate of blastocyst formation was increased (*P* < 0.05) in T2 (24.79%), T3 (21.43%), and T4 (18.52%), compared to T1 (2.89%). However, the rates of blocked embryos at the morula stage were 8.68%, 4.62%, 10.08%, and 8.23% in T1, T2, T3, and T4, respectively. The expression of genes regulating the antioxidant response (CAT, SOD1, and TXN), anti-apoptosis (BCL2), and metabolic activity (GLUT1) was upregulated in the treated groups.

**Conclusions:**

To conclude, the findings of this study clearly illustrate increased cleavage and blastocyst rates with supplementation of ascorbic acid, cysteine, or a combination of both. Furthermore, gene expression data support the positive effects of antioxidant supplementation to IVC media on enhancing embryonic development by promoting the intracellular defense mechanism and inhibition of apoptosis.

## Introduction

Assisted reproductive techniques (ARTs) have been relatively slower in dromedary camels compared to other domestic animal species. The characteristics of camels as a well-adapted, highly producing animal under harsh conditions, as well as the high place in the tribal culture for this animal in Middle Eastern countries, in addition to the increasing demand for camel milk, have sparked attention to camel breeding and research, such as in vitro embryo production (IVP). Several studies were conducted to investigate the factors affecting IVP outcomes in dromedary camels, including the methods and techniques of the IVP process, culture conditions, and media [1].

The primary objective of modern reproductive techniques, such as IVP, is to acquire the maximum quantity of high-quality embryos that can enable pregnancy induction after being transferred to recipient females. Few IVP studies have been successfully carried out in dromedary camels, albeit with low blastocyst rates ranging from 4% to 28% [2–4]. This embryo development rate is considered low, comparable to other species, for instance, cattle, which can achieve a blastocyst rate of up to 48% [5]. During the last few decades, numerous studies have indicated that the quality of the oocytes from which the embryos are produced directly influences their developmental potential [6, 7].

Moreover, there is reason to believe that the culture conditions of embryos impact their quality [8, 9]. Various factors affect the cultural environment, including medium content, protein addition, the number of embryos co-cultured, in addition to the levels of gases. During aerobic metabolism, reactive oxygen species (ROS) are produced naturally in the intermediary steps of oxygen reduction, even at the rest state. Furthermore, various environmental factors, such as protein oxidation, dead sperm cells, or metallic ions, might induce the generation of ROS by the embryo during IVC [10]. The embryo is exposed to OS when ROS accumulation exceeds the cellular capacity to generate antioxidants. Thus, the equilibrium between the availability of ROS and antioxidants is a vital determinant of successful embryonic development [11]. Embryos produced in vivo are shielded against OS by a combination of endogenous antioxidants and those that occur in the follicular and oviductal fluids [11, 12].

In contrast, the natural antioxidant defense mechanisms in IVC-embryos are insufficient to forestall OS; as a result, exogenous antioxidant supplementation could potentially be a vital solution [13]. Ascorbic acid and cysteine are both powerful antioxidants that aid cells in mitigating oxidative stress through distinct mechanisms. Ascorbic acid functions as a reactive oxygen species (ROS) scavenger in both in vivo and in vitro systems. It is a water-soluble vitamin that has long been acknowledged as the most crucial antioxidant present in extracellular fluid [14]. Cysteine, a precursor to glutathione, is the predominant endogenous intracellular antioxidant in mammalian cells. Furthermore, cysteine possesses a thiol group, which can undergo oxidation to form a disulfide derivative known as cystine [15]. The present study hypothesizes that using those antioxidants (ascorbic acid and cysteine) in IVC medium may decrease oxidative damage and improve in vitro embryo production outcomes.

Hence, this investigation aimed to study the impact of supplementing in vitro culture (IVC) medium with 150 µg/mL ascorbic acid, 100 µM cysteine, and a combination of both on the in vitro embryonic development and gene expression in dromedary camel embryos cultured under high oxygen concentration (20%).

## Materials and methods

### Chemicals and media

All chemicals and media constituents, unless specified otherwise, were procured from Sigma-Aldrich (St. Louis, MI, USA). Each of the media was prepared the night before using preserved stocks of ingredients, then purified before utilization as described by Russo et al. [16].

### Study location

The current work was carried out at the Embryology Manipulation Unit (EMU), Department of Animal and Poultry Physiology, Division of Animal and Poultry Production, Desert Research Center (DRC), Cairo, Egypt.

### Ovaries collection and oocytes recovery

**Fig. 1:**
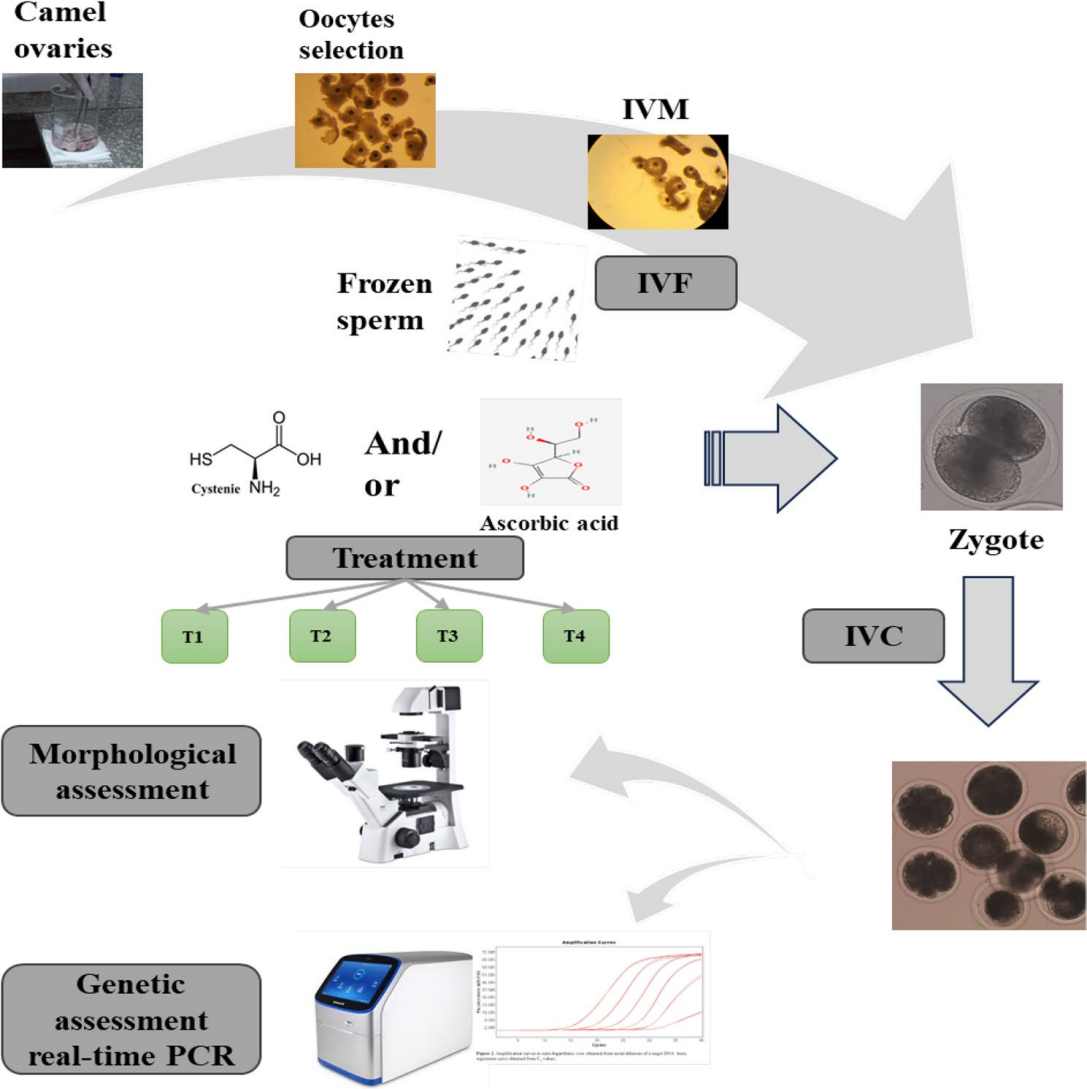
Showing an illustration of the experimental protocol. IVM: in vitro maturation. IVF: in vitro fertilization. IVC: in vitro culture. T1: Control. T2: 150 µg/mL ascorbic acid. T3: 100 µM cysteine. T4: 150 µg/mL ascorbic acid plus 100 µM cysteine

Post-slaughter of animals (*Camelus dromedarius*), ovaries were obtained from El-Bassatin abattoir, Cairo, Egypt. As illustrated in Fig. 1. Warm normal NaCl solution (0.9%), enriched via a combination of 100 IU penicillin and 100 µg/mL streptomycin, was used in transporting the ovaries to the lab. The frozen semen, stored in 0.5-mL straws, was acquired from the Artificial Insemination and Embryo Transfer Lab, Marriott Research Station, Desert Research Center (Alexandria, Egypt).

### IVM process

To extract cumulus-oocyte complexes (COCs) from the ovarian cortex, each ovary was sliced using a sharp razor-blade and rinsed via pre-warmed phosphate buffer saline (PBS) at 30 °C, plus gentamicin (50 µg/mL) [17, 18]. Retrieved COC-containing solution was inspected via stereomicroscope (GX microscope, GT Vision, Hagerstown, MD, USA) with a range of 8 to 50× to examine the COCs, and the COCs were morphologically evaluated following previous studies [17, 19]. In total, 961 high-quality COCs (class I and II, according to the IETS classification) were gathered and divided among the four study groups. All COCs were in vitro matured in the same conditions, the IVM medium consisted of TCM-199 plus 40 IU/mL pregnant mare serum gonadotropin (PMSG), 20 ng/mL epidermal growth factor (EGF), 15% (v/v) fetal bovine serum (FBS), 0.25 mg/mL sodium pyruvate, 1 µg/mL estradiol (E2), and 50 µg/mL gentamicin. Regarding the maturation drops, a number of 15 COCs per 100-µL drop were overlayered via mineral oil and incubated in a CO_2_ incubator for 30 h at 38.5 °C under 5% CO_2_, 20% O_2_, 75% N_2_, and 95% relative humidity [17, 19]. Every experimental group was repeated 5 times, each replicate contained around 45 to 50 of COCs.

### Semen preparation

Frozen semen thawing process was conducted at 37 °C for 40 s [20]. Following that, the content of two straws was overlayered by 5 mL of sperm washing medium, which consisted of Tyrode’s albumin lactate pyruvate-based solution (TALP) without calcium chloride. Afterward, the sperm were subjected to double centrifugation for 5 min each at 300 × *g*. The remaining pellet has been reconstituted using a suitable amount (estimated based on the concentration of the sperm after washing) of a pre-warmed medium fertilization medium (complete TALP medium supplemented with 0.6% bovine serum albumin (BSA), 1 mM sodium pyruvate, and 50 µg/mL gentamicin) for fertilization. Then, the number of sperm was set to 3 × 10^6^ sperm/mL [21].

### IVF process

At the end of the IVM step, all matured oocytes were rinsed with fertilization medium before being co-cultured with the prepared sperm in a CO_2_ incubator at 38.5 °C under 5% CO_2_, 20% O_2_, and 75% N_2_ for 18 h as indicated by Khattab et al. [21].

### Cumulus cells’ denudation

The presumptive zygotes were rinsed with wash medium (WM: TCM-199 plus 25 mM HEPES and 5% FBS), then kept for 2 min with 80 IU/mL hyaluronidase [22] in a FertiCult Flushing medium (Fertipro©, Beernem, Belgium). Succeeded with repeated pipetting of the oocytes in the WM to get rid of any attached cumulus cells and sperm.

### In vitro culture

Following denudation, the presumed zygotes were carefully washed via an IVC medium comprised of TCM-199 [23] plus 50 µg/mL gentamicin, 5% FBS, 1X MEM essential amino acids, 1X MEM nonessential amino acids. According to the experimental group, various amounts of ascorbic acid and cysteine were applied to the IVC media. After that, embryos were incubated at 38.5 °C in a humid atmosphere (5% CO_2_, 20% O_2_, and 75% N_2_) in air for 6 d. According to previous studies conducted in our lab on ascorbic acid supplementation of IVC media [19] and cysteine concentrations in camels [24], the IVC media of dromedary camel embryos in the current investigation was enriched with antioxidants as follows: control group without antioxidant supplementation (T1), 150 µg/mL ascorbic acid (T2), 100 µM cysteine (T3), and a combination of both antioxidants (150 µg/mL ascorbic acid and 100 µM cysteine) (T4). The IVC media renewal occurred every 48 h by substitution 50% of the culture well volume with fresh medium of the same type, to replenish nutrients and remove the toxic metabolites. Embryonic development was examined via an inverted microscope (Leitz Fluovert FU; Leica Microsystems, Wetzlar, Germany). The cleavage rate was monitored at 48 h, and the morula and blastocyst rates were calculated at 6 d post-fertilization.

## Gene expression

### RNA extraction and cDNA synthesis

These steps were carried out utilizing three separate replicates from each experimental group (two embryos [at the blastocyst stage] per replicate). Total RNA was isolated using the PicoPureTM RNA Isolation Kit (MDS Analytical Technologies GmbH, Ismaning, Germany) in accordance with the manufacturer’s instructions. A lysis buffer was added to the embryos, and they were then kept for 30 min at 42 °C in a Thermo Block. To enable the RNA to attach to the spin column, the entire lysis mixture was put onto a pre-conditioned spin column and centrifuged for a brief period of two minutes at 3600 × g. The DNA contaminants were eliminated via the RNAse-Free Dnase Set (Qiagen GmbH, Hilden, Germany). Two distinct wash buffers (WB1 and WB2) were used to wash the column twice. Furthermore, 16 µL of RNAse-Free Water (Qiagen GmbH) was used to elute the RNA.

The High-Capacity cDNA Reverse Transcription Kit (Life Technologies, Carlsbad, CA, USA) was used to synthesize cDNA for each sample. The final RNA amount of each sample was edited via Water (Rnase-Free) to attain an equal concentration of the RNA. Next, a volume (14.2 µL) of RNA sample was incorporated to 1 µL 10× RT buffer, 0.8 µL 25× dNTP, 2 µL random primer, 1 µL RNAse inhibitor, and 1 µ1 MultiscribeTM reverse transcriptase, and the mixture was incubated at 25 °C for 10 min, 37 °C for 120 min, and 85 °C for 5 min and held at 4 °C. Before being quantified using real-time PCR, the cDNA samples were kept at −20 °C.

### Real-time PCR analysis

Six genes [BCL2, CAT, CuZn-SOD (SOD1), GLUT1 (SLC2A1), p53, and TXN] were chosen based on their biological functions during preimplantation embryo development. The primers (Table 1) were designed with the aid of the Primer3 software (http://primer3.wi.mit.edu/) and the housekeeping gene GAPDH was used as a reference gene. The mixture of the PCR reaction contained 12.5 µL Maxima SYBR Green PCR Master Mix (Thermo Fisher Scientific, Waltham, MA, USA), 0.2 µL forward primer, 0.2 µL reverse primer, and 5.1 µL nuclease-free water, added to 2 µL cDNA. The thermal cycling protocol consisted of an initial denaturation step at 50 °C for 2 min, 95 °C for 10 min, followed by 40 cycles of amplification (95 °C for 15 s, 60 °C for 20 s, 72 °C for 30 s). A final extension step was performed at 60 °C for 1 min. Subsequently, a melt curve analysis was conducted by gradually increasing the temperature from 60 °C to 95 °C, acquiring fluorescence data every 7 s. The relative expression was determined by applying the method of relative quantification (2^−ΔΔCT^ method) according to Bermejo-Alvarez et al. [25].

**Table 1:** Primers utilized for quantitative real-time PCR evaluation

Name	GenBank accession number	Primer sequence	Fragment size (bp)
CuZn-SOD (SOD1)	JF758876.1	F: 5′- TGCAGGCCCTCACTTTAATC-3′ R: 5′-CTGCCCAAGTCATCTGGTTT-3′	216
GLUT1	XM_010982338.2	F: 5′- CTTGCCTGAGACCAGTTGGG-3′ R: 5′-TTACAGAAGGAGTAAGGCGGTG-3′	296
p53	XM_010996514.2	F:5′- CCACCTGAAGTCTAAGAAGG-3′ R:5′- AGTGCAGGTCAACTTCTTTA-3′	250
BCL2	XM_010979993.2	F: 5′- ACATCCACTATAAGCTGTCG3′ R: 5′- TAGCGCCGAGAGAAGTCAT3′	241
TXN	XM_010978718.2	F: 5′- TGATCAAGCCTTTCTTTCAT-3′ R: 5′- TAATGGTGGCTTCAAGTTTT-3′	195
CAT	XM_011000575.2	F: 5′-GAAACGCCTGTGTGAGAAC-3′ R :5′-ACATAGGTGTGAACTGCGT-3′	142
GAPDH	XM_010990867.2	F: 5′- AGGTCGGAGTGAACGGATTC-3′ R: 5′- GGAAGATGGTGATGGCCTTT-3′	219

*F* Forward cDNA strand, *R* Reverse cDNA strand, *bp* base pair

### Statistical analysis

Data of embryonic development were assessed with Chi-square analysis and were presented as a percentage (%). In addition, a data transformation using the arcsine transformation method (arcsin(sqrt(x)), where X indicates the percent value) was conducted, then a one-way ANOVA test was applied to find the variation between the experimental groups. Comparisons were significantly different if *P* < 0.05. Concerning gene expression analysis, the general linear model approach was utilized to statistically analyze the data (SAS, 2008), which was presented as mean ± standard error of the mean. To define significance, a *P* < 0.05 was used.

## Results

In this experiment, embryonic development data displayed in Table 2 showed that rates of cleavage were increased (*P* < 0.05) in groups supported with 150 µg/mL ascorbic acid (T2: 29.41%), 100 µM cysteine (T3: 32.77%), or a combination of both (150 µg/mL ascorbic acid and 100 µM cysteine) (T4: 27.16%) in contrast to the control group (T1: 14.05%). However, no significant differences were noticed across groups that were treated with antioxidants regardless of the antioxidant type or combination. Also, the data in Table 2 indicated that the rates of blocked embryos at the morula stage were 8.68%, 4.62%, 10.08%, and 8.23% in the control (T1), ascorbic acid (T2), cysteine (T3), and combination (T4) groups, respectively. These findings indicated that ascorbic acid treatment significantly reduced the number of blocked embryos at the morula stage compared to all other groups. On the other hand, supplementing the IVC medium with ascorbic acid and/or cysteine (T2, T3, and T4) resulted in a higher blastocyst rate (*P* < 0.05) compared to the control group (24.79%, 21.43%, and 18.52% vs. 2.89%, respectively) as shown in Table 2. Notably, the T2 group was significantly higher than the T3 and T4 groups, while the T4 group was significantly lower than the T3 group.

**Table 2:** Effect of incorporating antioxidants to IVC medium on the early embryonic development in dromedary camel

Treatment	COCs (*n*)	Cleavage rate 48 hpi (%)	Blocked morula (%)	Blastocyst rate (%)
T1	242	14.05 ^d^ (34/242)	8.68 ^a^ (21/242)	2.89 ^d^ (7/242)
T2	238	29.41 ^b^ (70/238)	4.62 ^b^ (11/238)	24.79 ^a^ (59/238)
T3	238	32.77 ^a^ (78/238)	10.08 ^a^ (24/238)	21.43 ^b^ (51/238)
T4	243	27.16 ^c^ (66/243)	8.23 ^a^ (20/243)	18.52 ^c^ (45/243)
Chi value		25.35	5.31	48.38

^a and b^: Superscript letters within the same column to be compared statistically

Values with different letter superscripts have significant differences at (*P* < 0.05). COCs, cumulus-oocyte complexes; hpi, hours post insemination. T1: Control. T2: 150 µg/mL ascorbic acid. T3: 100 µM cysteine. T4: 150 µg/mL ascorbic acid plus 100 µM cysteine

**Fig. 2:**
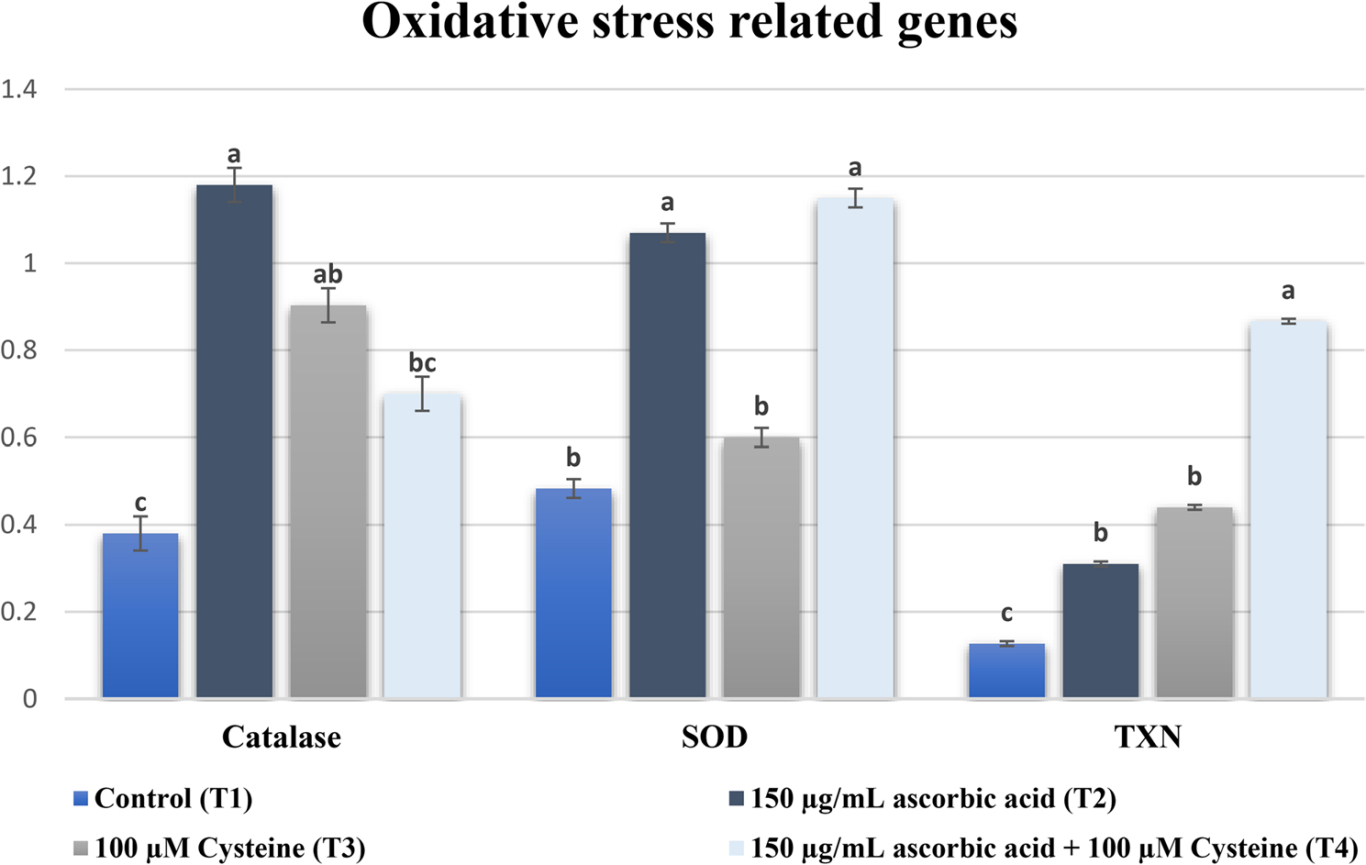
Impact of incorporating antioxidants to IVC medium of dromedary camel embryos on the expression profile of the oxidative stress related genes; Catalase, Superoxide dismutase (SOD), and thioredoxin (TXN). Bars marked with letters a, b and c are significantly different (*P* < 0.05). Sample size (*n* = 6 blastocyst/group)

**Fig. 3:**
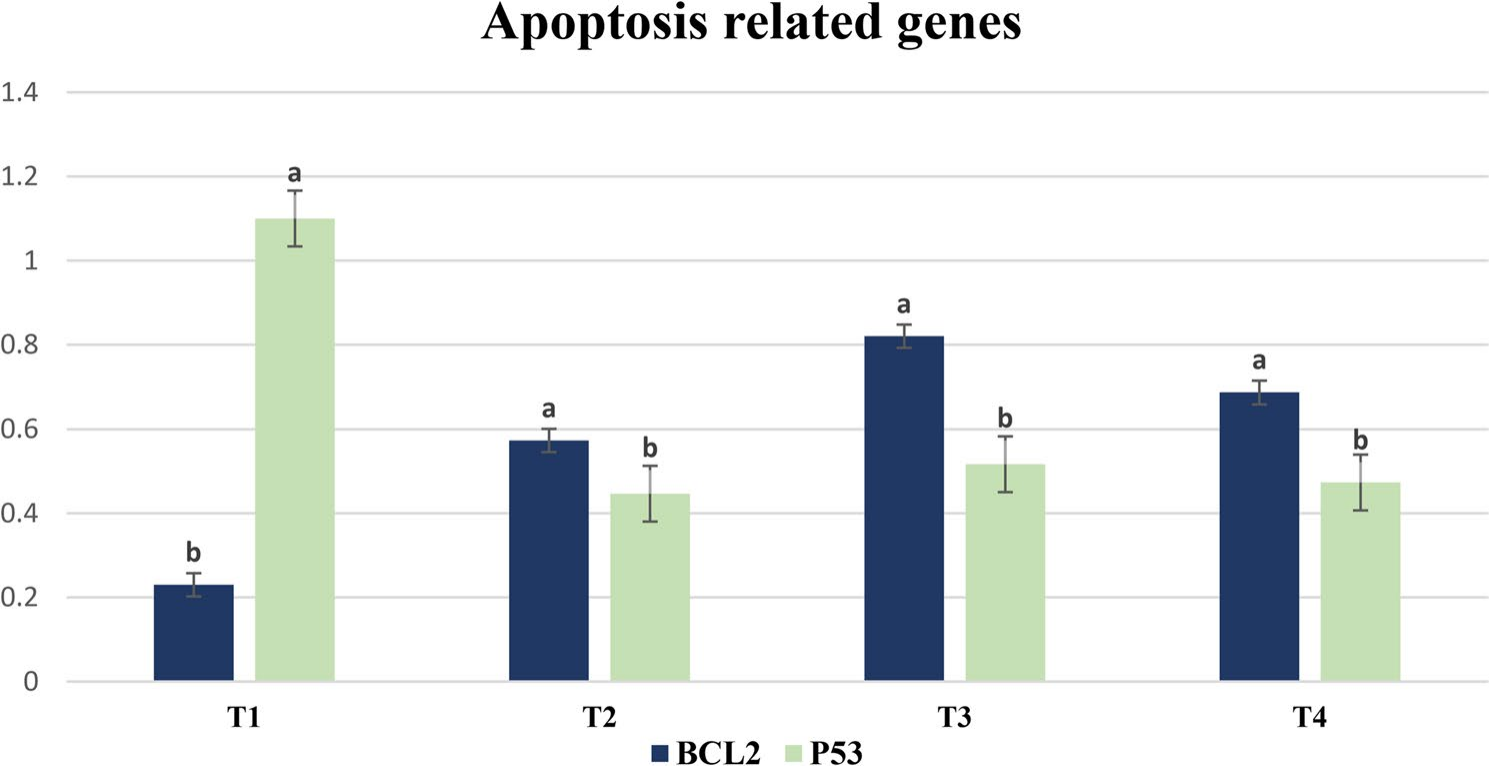
Impact of incorporating antioxidants to IVC medium of dromedary camel embryos on the expression profile of the apoptosis related genes (BCL2 and p53). T1: Control. T2: 150 µg/mL ascorbic acid. T3: 100 µM cysteine. T4: 150 µg/mL ascorbic acid plus 100 µM cysteine. Bars marked with letters a and b are significantly different (*P* < 0.05). Sample size (*n* = 6 blastocyst/group).

**Fig. 4:**
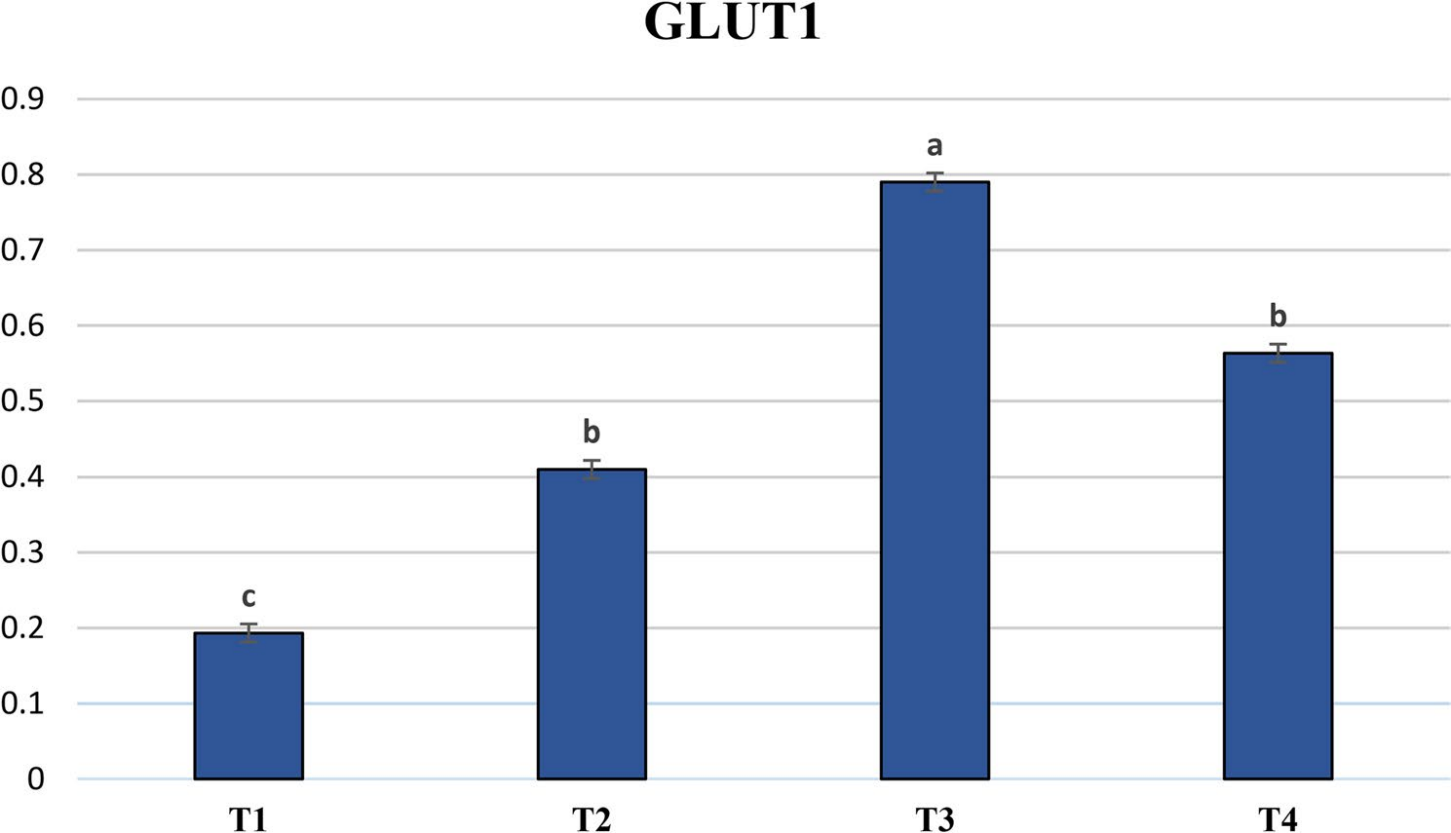
Impact of incorporating antioxidants to IVC medium of dromedary camel embryos on the expression profile of the GLUT1 gene. T1: Control. T2: 150 µg/mL ascorbic acid. T3: 100 µM cysteine. T4: 150 µg/mL ascorbic acid plus 100 µM cysteine. Bars marked with letters a, b and c are significantly different (*P* < 0.05). Sample size (*n* = 6 blastocyst/group)

The expression analysis of oxidative stress related genes (Fig. 2), apoptosis related genes (Fig. 3), and metabolism related gene (Fig. 4) showed that catalase was significantly upregulated in the single-antioxidant treatment groups (T2 and T3) relative to the control group. Furthermore, there were no differences (*P* < 0.05) between these two groups. On the other hand, the combined antioxidant treatment (T4) group demonstrated no difference (*P* < 0.05) compared to either the cysteine (T3) or the control (T1) groups, regardless of the upregulation that took place in the T4 group compared to the control group. The data obtained from GLUT-1 gene expression analysis demonstrated upregulation in all antioxidant-treated groups compared to the control group. However, there was an increase (*P* < 0.05) in GLUT-1 gene expression in the cysteine group (T3) compared to both the ascorbic (T2) and ascorbic + cysteine (T4) groups. The expression profile of the antioxidant-related gene SOD1 was reduced (*P* < 0.05) in both T1 and T3 groups compared to the T2 and T4 groups. The SOD1 expression level was higher in the T3 group compared to the T1 group; however, this increase was not statistically significant. The expression of TXN was increased (*P* < 0.05) in embryo groups supplemented with one of the antioxidants or a combination of both compared to the control group. Moreover, TXN expression was higher (*P* < 0.05) in the combined antioxidant (T4) group than in either of the single-antioxidant groups (T2 and T3). The transcriptional profile of p53, the apoptosis-inducing gene, was higher (*P* < 0.05) in the control group than in all antioxidant-treated groups. Meanwhile, the expression of BCL2, the anti-apoptosis gene, was higher (*P* < 0.05) in all antioxidant-treated groups in comparison with the control.

## Discussion

The results of the current study revealed that the supplementation of ascorbic acid, cysteine, or a combination of both antioxidants improved cleavage and blastocyst development of IVP camel embryos. The ascorbic acid (150 µg/mL) supplemented group (T2) showed a significant increase in blastocyst formation rate, while the cysteine (100 µM) group (T3) produced the highest (*P* < 0.05) cleavage rate compared to the control (T1). The combined group (T4) showed a significantly higher cleavage and blastocyst rates compared to the control. However, it was significantly lower than the T2 and T3. In addition, the findings of gene expression analysis supported the embryonic development results.

These findings concur with research by Wang et al. [25] that found that mouse embryos exposed to ROS for an extended period of time experienced embryotoxicity, and that subjecting these embryos to ascorbic acid at a 50 µM concentration markedly accelerated the formation of blastocysts. Similarly, Kere et al. [26] found that adding 50 µg/mL of ascorbic acid to media during IVP of porcine embryos improved the cleavage and blastocyst rates, in addition to the total cell number per embryo. Such findings might be attributed to ascorbic acid administration during IVC, which boosted intracellular levels of glutathione (GSH) while decreasing ROS accumulation. Additionally, it was reported [27] that the incorporation of ascorbic acid into the media during the IVP process improved the embryonic development in buffalo. Conversely, these results are at odds with those [28] reported that supplementation with ascorbic acid at a 50 µg/mL concentration showed no discernible influence on both IVM and IVF, in addition to the developmental competence of embryos in porcine. However, they also stated that the inclusion of ascorbic acid promoted the cryo-survival rate of IVP blastocysts following vitrification. Indeed, supplementation of antioxidants would be one of the strategies to support the establishment of a new IVP protocol for camels.

Although cysteine (6.9 µM) addition to the maturation medium has been demonstrated to positively affect the rate of IVM based on the expansion of cumulus cells [21], there are currently no available studies examining the impact of cysteine supplementation during IVC of dromedary camel embryos. The enhanced camel embryo development observed when embryos were exposed to different antioxidants was coupled with suppressed expression of genes corresponding to the induction of OS, as well as apoptosis, for instance, p53. This hypothesis is supported by a study [25] that indicated that ROS levels have decreased due to supplementation via antioxidants that downregulated p53 expression in mouse embryos. Notably, the upregulation of the p53 gene increases the cellular death rate during severe OS, thereby halting the progression of oxidative chain damage and preventing its spread to neighboring cells by killing the affected cells [29–31]. In addition, the upregulation of the BCL2 gene inhibits the apoptosis process [32]. Moreover, Yang and Rajamahendran [33] observed a positive correlation between BCL2 expression levels and embryo quality in bovine, as higher expression was detected in good-quality embryos compared to bad-quality embryos. Taken together, the aforementioned data indicate that antioxidant supplementation promotes embryo development by upregulating anti-apoptotic genes and downregulating pro-apoptotic genes, which may suggest an improvement in embryo quality. To our knowledge, gene expression data of this investigation is the first performed in IVP produced camel embryos, which supports ongoing efforts to identify molecular fingerprints of this unique species.

Additionally, El-Naby et al. [27] reported that combined antioxidant incorporation into the media through the IVM and IVC steps yielded positive effects on the IVP of buffalo embryos [27]. Moreover, Córdova et al. [34] showed that 5 µL/mL insulin-transferrin-selenium (ITS) plus 100 µg/mL ascorbic acid addition through the first 12 h of IVM improved cytoplasmic maturation in bovine embryos derived from prepubertal oocytes. Indeed, under certain circumstances, ascorbic acid has been demonstrated to operate synergistically with α-tocopherol (vitamin E) [35]. However, Hossein et al. [36] reported that both the rate of porcine blastocyst development and the number of cells within it were not affected by ascorbic acid solely or in combination with α-tocopherol. This suggests that supplementation of different combined antioxidants may have no positive effect or even lead to a negative impact on IVP. For example, Olson and Seidel [37] reported that combining α-tocopherol and ascorbic acid at a concentration of 100 µM each in bovine embryos had a negative impact on embryo development rate. In addition, when α-tocopherol and ascorbic acid were combined in the IVM medium for bovine oocytes, they reduced the number of blastocysts significantly in comparison to the control, α-tocopherol, and ascorbic acid independently [38]. Taken together, it is suggested that the IVC culture medium be supplemented with a single antioxidant in order to enhance IVP in camels. This agrees with the results of the current study, which showed that the addition of combined antioxidants (150 µg/mL ascorbic acid plus 100 µM cysteine) had a significant positive effect on the embryonic development of in vitro cultured camel embryos compared to the control group. However, the results also demonstrated that the single administration of ascorbic acid (150 µg/mL) or cysteine (100 µM) had a more positive effect compared to the combination of both.

According to reports [39, 40], cells have the ability to increase the catalase levels of expression in response to an increase in ROS concentrations as a mechanism to counteract OS. Likewise, a number of studies revealed that the expression of the catalase gene is downregulated when the oxygen concentration during embryo IVC is reduced, leading to low ROS concentrations [41–43]. Alternatively, when embryos were exposed to increased ROS levels, this gene was upregulated [44, 45]. Our results differ from these previously mentioned findings, as we observed an upregulation of the catalase gene in antioxidant-treated embryos. This could be attributed to the possibility that the antioxidant treatment increases the expression of catalase, enhancing the embryo’s defense mechanisms to neutralize ROS produced under high oxygen tension. Our findings are consistent with the work conducted by Rocha-Frigoni et al. [46], who indicated a reduction in ROS level and apoptosis rate within the embryos cultured in vitro with medium enhanced via antioxidants (catalase, cysteine, and β-mercaptoethanol) in bovine.

The results of GLUT-1 expression suggested that supplementing IVC medium with antioxidants improved glucose consumption and utilization by IVP camel embryos. This finding is supported by the study conducted by Lim et al. [47], who reported that GLUT-1 gene expression was significantly increased in bovine embryos developed in defined media compared to undefined media. This improvement could be due to antioxidant-induced alleviation of OS in the culture medium. Evidence from the study of Kind et al. [48] lends support to this hypothesis; they demonstrated a 2- to 4-fold increase in GLUT-1 expression in embryos cultured under 2% oxygen as compared to those cultured under 20 or 7% oxygen. However, variations in GLUT-1 gene expression patterns could occur based on the species, as demonstrated in a study done by Rajhans et al. [49], which reported the absence of the GLUT-1 gene in buffalo embryos after the eight to 16-cell stages. This finding is in contrast to the reported upregulation of this gene in mouse embryos from the two-cell to the blastocyst stage [50]. Notably, the data of the present study highlight the dynamic nature of IVP embryos’ gene expression among mammalian species.

In a recent study by Ghanem et al. [51], the candidate marker gene SOD2 was upregulated in bovine embryos developed in IVC medium supplemented with different supplements (l-carnitine, phenazine ethosulfate, and l-carnitine + phenazine ethosulfate). Indeed, SOD1 and SOD2 are two members of the gene family expressed in mitochondria, and their upregulation reflects both the increased metabolic activity of mitochondria [50] and the ability of embryos to scavenge ROS [52]. Consequently, they are regarded as molecular markers of competent embryos [53]. The expression profiles of CuZn-SOD (SOD1) and Mn-SOD (SOD2) were detected at all preimplantation stages of mouse embryos produced either in vitro or in vivo [54]. Additionally, the protein of the two enzymes was abundant in all stages of rat early development, indicating their critical roles in mitigating OS, and surprisingly, both were abundant in the cytoplasm, either as diffused in the whole cytoplasm (SOD1) or as small clusters (SOD2) [55]. Noteworthy, bovine embryos produced in vitro expressed mRNAs for SOD1, whereas expression of SOD2 was not detected at any preimplantation stage [54]. As well-established by recent investigations [56–59], the signaling pathway of NRF2 includes numerous candidate genes, and the expression profile of NRF2 seems to be linked with the abundance of SOD1 and SOD2 transcripts. However, attenuation of NRF2 mRNA significantly resulted in downregulation of SOD2 expression [59]. This implies that enriching IVC media via ascorbic acid alone or ascorbic acid + cysteine could support the development of camel embryos by increasing metabolic activity and improving the intercellular microenvironment, which is regulated by SOD family genes. Our hypothesis was further supported by a study reporting the upregulation of SOD2 in bovine blastocysts that developed after exogenous PRDX II was added to an IVM medium, indicating a beneficial effect on mitochondrial function [60]. Taking into consideration that the previous investigation [57] was conducted using a limited sample size (three embryo biopsies) for RNA isolation, which is a similar procedure to that applied in the present study, which confirms high gene expression reliability.

Additionally, TXN gene expression was upregulated in both in vitro- and in vivo-derived bovine blastocysts, ultimately resulting in successful pregnancy [61]. The expression profile of TXN increased in cytotrophoblast cells during embryo implantation [62]. The transcript of this gene is well-known as an early pregnancy factor [63, 64]. This gene is a key regulator of various biological processes, such as cell proliferation [65] and anti-apoptotic function [66]. It should be noted that TXN helps the developing embryo to resist OS under in vitro conditions, as shown in an investigation done in mice [67]. Moreover, it has been reported that supplementing the culture medium with thioredoxin leads to increased development rate and total cell numbers as well as reduced apoptotic cell numbers in mice [68], bovine [69], and swine embryos [70]. Therefore, it seems that the supplementation of one of the antioxidants alone or combined has upregulated TXN in camel embryos to provide favorable intercellular conditions for in vitro development. The upregulation of antioxidant genes is induced in embryonic cells to protect against apoptosis, which is a key biological process that can reduce embryo quality and impair the ability to establish pregnancy [51, 61]. Overall, the addition of antioxidants to IVC medium provides intracellular protection against OS in developing embryos, which is a prerequisite for subsequent in vitro development until blastocyst formation.

## Conclusions

Supplementation of IVC medium with ascorbic acid, cysteine, or a combination of both positively impacted the development rate of in vitro-produced camel embryos. These beneficial effects of antioxidants supplementation are likely a result of the protective mechanism against ROS accumulation and apoptosis, as evidenced by the molecular genetic data of selected genes correlated with OS, apoptosis, and embryo metabolism.

## Data Availability

The datasets generated during and/or analyzed during the current study are available from the corresponding author upon reasonable request.
